# Has universal screening with Xpert® MTB/RIF increased the proportion of multidrug-resistant tuberculosis cases diagnosed in a routine operational setting?

**DOI:** 10.1371/journal.pone.0172143

**Published:** 2017-02-15

**Authors:** Pren Naidoo, Rory Dunbar, Judy Caldwell, Carl Lombard, Nulda Beyers

**Affiliations:** 1 Desmond Tutu TB Centre, Department of Paediatrics and Child Health, Faculty of Medicine and Health Sciences, Stellenbosch University, Stellenbosch, South Africa; 2 City Health Directorate, Cape Town, South Africa; 3 Biostatistics Unit, South African Medical Research Council, Cape Town, South Africa; McGill University, CANADA

## Abstract

**Setting:**

Primary health services in Cape Town, South Africa where the introduction of Xpert^®^ MTB/RIF (Xpert) enabled simultaneous screening for tuberculosis (TB) and drug susceptibility in all presumptive cases.

**Study aim:**

To compare the proportion of TB cases with drug susceptibility tests undertaken and multidrug-resistant tuberculosis (MDR-TB) diagnosed pre-treatment and during the course of 1st line treatment in the previous smear/culture and the newly introduced Xpert-based algorithms.

**Methods:**

TB cases identified in a previous stepped-wedge study of TB yield in five sub-districts over seven one-month time-points prior to, during and after the introduction of the Xpert-based algorithm were analysed. We used a combination of patient identifiers to identify all drug susceptibility tests undertaken from electronic laboratory records. Differences in the proportions of DST undertaken and MDR-TB cases diagnosed between algorithms were estimated using a binomial regression model.

**Results:**

Pre-treatment, the probability of having a DST undertaken (RR = 1.82)(p<0.001) and being diagnosed with MDR-TB (RR = 1.42)(p<0.001) was higher in the Xpert-based algorithm than in the smear/culture-based algorithm. For cases evaluated during the course of 1st-line TB treatment, there was no significant difference in the proportion with DST undertaken (RR = 1.02)(p = 0.848) or MDR-TB diagnosed (RR = 1.12)(p = 0.678) between algorithms.

**Conclusion:**

Universal screening for drug susceptibility in all presumptive TB cases in the Xpert-based algorithm resulted in a higher overall proportion of MDR-TB cases being diagnosed and is an important strategy in reducing transmission. The previous strategy of only screening new TB cases when 1st line treatment failed did not compensate for cases missed pre-treatment.

## Introduction

Multidrug-resistant tuberculosis (MDR-TB), defined as resistance to rifampicin and isoniazid, presents a major global health challenge with an estimated 480,000 incident cases in 2014 [1]. Whilst MDR-TB rates are substantially higher among previously treated cases (20%) than among new cases (3.3%), the latter contribute substantially to the total burden, accounting for an estimated 53% of MDR-TB cases among the pulmonary TB cases notified in 2014 [1]. The high proportion of new TB cases with MDR-TB (primary MDR-TB) suggests significant disease transmission.

Persistent diagnostic gaps due to both inadequate TB case-detection and limited drug susceptibility testing among detected cases contribute to transmission of MDR-TB. Only 6 million of the estimated 9.6 million TB cases in 2014 were detected. Among notified pulmonary TB cases, 300,000 were estimated to have MDR-TB; however just 12% of new and 58% of previously treated cases were tested for drug susceptibility and 123,000 MDR-TB cases were notified globally [1]. Thus it is estimated that 180,000 of the missed MDR-TB cases were among those with undetected TB and 177,000 among detected TB cases not screened for drug susceptibility.

The past reliance on insensitive smear microscopy tests [2–4] as the cornerstone of laboratory diagnosis has contributed to poor TB case-detection, particularly among human immunodeficiency virus (HIV)-infected cases, where sensitivity can be as low as 23% to 50% [5–9]. Limited laboratory capacity for culture and drug susceptibility testing (DST) contributes to poor MDR-TB case-detection: among the 36 high TB or MDR-TB burden countries, only 16 met the benchmark of one laboratory with culture and DST capabilities per five million population in 2010 [10]. In many developing countries these limitations have led to DST being rationed for use among previously treated cases as they are most at risk of MDR-TB [11–13].

The availability of the more sensitive Xpert^®^ MTB/RIF (Xpert)(Cepheid, Sunnyvale, CA, USA) test offers the possibility of improving TB case-detection and simultaneously screening for drug susceptibility. Xpert is a nucleic acid amplification test that detects genetic sequences for *Mycobacterium tuberculosis* (MTB) complex and simultaneously, the presence of ‘wild type’ or mutations conferring resistance to rifampicin. Xpert has the ability to detect low bacteria loads (limit of detection 131 colony forming units (CFU) per ml) [14] compared to 10,000 CFU per ml for smear [15]) and is particularly useful in diagnosing the smear-negative TB typically found in HIV-infected individuals. Xpert has high sensitivity and specificity for detecting both TB and rifampicin resistance. In a meta-analysis of fifteen studies where Xpert was used as the initial test replacing smear microscopy, pooled sensitivity was 88% (95%CrI 83% to 92%) and specificity was 98% (95% CrI 97% to 99%) for detecting MTB. In eleven of these studies, pooled sensitivity was 94% (95% CrI 87% to 97%) and specificity was 98% (95% CrI 97% to 99%) for rifampicin resistance [16].

While Xpert has the technical capacity to help close MDR-TB diagnostic gaps, very little has been reported on its use in universal drug susceptibility screening under routine operational conditions. A study in India found that routine, up-front use of Xpert in children under 14-years of age almost tripled the number of Rif-R cases diagnosed (from 22 to 60 among the 8,370 presumptive TB cases screened), than would have been achieved through screening of presumptive DR-TB cases only [17].

Our study compared the proportion of TB cases with DST undertaken and MDR-TB diagnosed pre-treatment in the previous smear/culture-based algorithm and the newly introduced Xpert-based algorithm. Among cases without MDR-TB diagnosed pre-treatment, we compared the proportion with DST undertaken and MDR-TB diagnosed during the course of 1^st^-line TB treatment. Comparisons were made for new and previously treated TB cases. The study was part of PROVE IT (Policy Relevant Outcomes from Validating Evidence on ImpacT), an evaluation to assess the impact of new molecular diagnostics on TB and MDR-TB diagnosis and treatment initiation in a routine operational context.

## Methods

We had previously undertaken and published results of a non-randomised stepped-wedge study to assess TB yield [18]. The current study assessed drug susceptibility screening and MDR-TB diagnosis among the TB cases identified in the previous study. Some details are highlighted here for completeness.

### Setting

**Fig 1:**
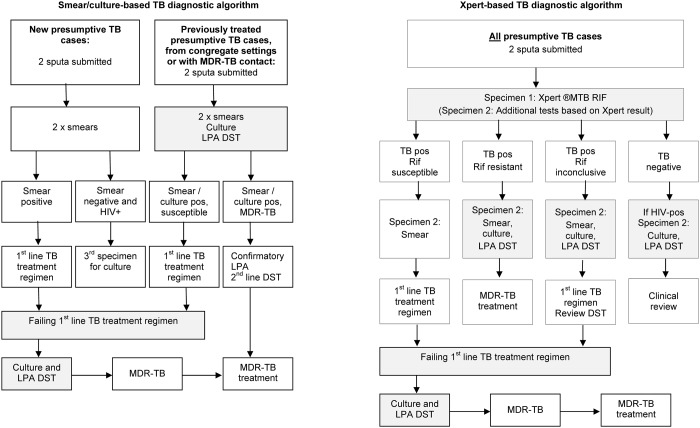
Testing in the smear/culture and Xpert-based TB diagnostic algorithms. The sequence of diagnostic tests in each algorithm and the action taken based on test results is shown. Shaded blocks indicate possible MDR-TB diagnostic points. Abbreviations: MDR-TB—multidrug-resistant tuberculosis; LPA—GenoType^®^ MTBDRplus line probe assay; DST—drug susceptibility testing; HIV–human immunodeficiency virus; Rif–rifampicin; Pos–positive.

The study was undertaken in Cape Town, South Africa where free TB diagnostic services were provided at 142 primary health care (PHC) facilities in eight health sub-districts. Prior to August 2011, a smear/culture-based diagnostic algorithm was used (Fig 1). All presumptive TB cases were evaluated through two spot sputum specimens, taken 1-hour apart and examined by fluorescence microscopy. In previously treated TB cases, those from congregate settings or with an MDR-TB contact, the second specimen also underwent liquid culture (BACTEC^™^ MGIT^™^ 960) and DST with GenoType^®^ MTBDRplus (Hain LifeScience GmbH, Nehren, Germany) line probe assay (LPA).

Between August 2011 and February 2013 an Xpert-based algorithm was phased into the eight health sub-districts with Xpert replacing smear microscopy for *all* (new and previously treated) presumptive TB cases (Fig 1). The first of two sputum specimens submitted was tested with Xpert; if rifampicin resistance was detected, the second specimen underwent smear, culture and LPA.

In both algorithms, new and previously treated cases in which first line TB treatment regimens failed had specimens submitted for culture and LPA during the course of treatment.

Routine TB Programme data collected during the study period, indicated that the number of notified TB cases declined from 28,644 in 2011 (752/100,000 population) to 25,846 in 2013 (663/100,000 population). HIV co-infection rates were 47% (97% tested) and 44% (98% tested) in respective years. The number of MDR-TB cases notified increased from 1,020 to 1,134, comprising 3.6% and 4.4% of TB cases respectively (Routine TB Programme Data, Cape Town Health Directorate, April 2016).

### Definitions

*A presumptive TB case* was defined as an individual with pre-treatment sputum samples submitted for diagnostic purposes.

*A TB case* was defined as an individual with one or more smears positive and / or culture positive for MTB and / or MTB detected on Xpert. TB cases were categorised as *new* (an individual with no or less than four weeks of previous TB treatment) or *previously treated* (an individual with more than four weeks of previous TB treatment).

*An MDR-TB case* was defined as a TB case with rifampicin resistance on LPA or Xpert, diagnosed either pre-treatment or whilst on 1^st^ line TB treatment. Rifampicin resistance was used as a proxy indicator of MDR-TB.

### Study design, timeframes and population

**Fig 2:**
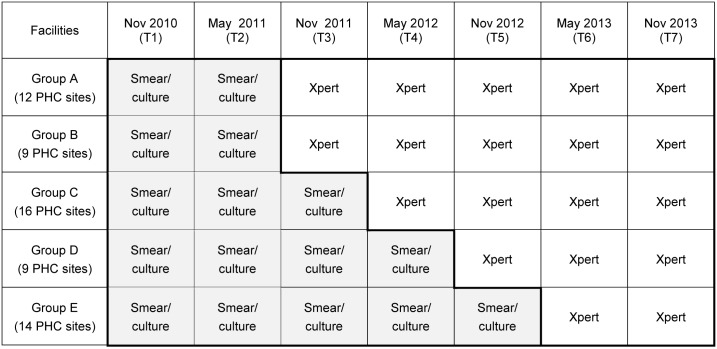
TB cases from the stepped-wedge analysis of TB yield included in this analysis. This figure shows the TB diagnostic algorithm in place in 5 groups of PHC sites over seven time-points (T1 to T7) during the transition from the smear/culture-based algorithm to the Xpert-based algorithm. All TB cases identified in the smear culture-based algorithm (T1 to T5) and in the Xpert-based algorithm (T3 to T7) were included in the current analysis. A PHC site consisted of municipal and provincial health facilities linked to their satellite and mobile facilities and to each other if within a single geographic location (to account for shared diagnostic services). Abbreviations: PHC = primary health care.

The previously published stepped-wedge study assessed TB yield in five sub-districts over seven one-month time-points (T1 to T7) prior to, during and after the introduction of the Xpert-based algorithm [18]. The current study population included all presumptive TB cases with a bacteriological diagnosis of TB in the smear/culture-based algorithm (T1 to T5) and in the Xpert-based algorithm (T3 to T7) (Fig 2).

### Data sources & management

The National Health Laboratory Services provided TB test data from the electronic laboratory database for 2010–2014. Data included clinic identification, patient clinical folder number, patient demographics, TB category (new, previously treated), test type (smear, culture, LPA or Xpert), diagnostic point (pre-treatment or on 1^st^-line treatment), test date and result.

Laboratory data for all primary health facilities and all hospitals in Cape Town were imported into Microsoft SQL. We used a combination of patient name, surname, birth date, address and clinical folder number to identify all DSTs undertaken (i.e. a rifampicin result on Xpert / LPA) for the TB cases diagnosed at T1 to T7 and to identify duplicates cases (individuals diagnosed at multiple facilities). DSTs were classified as pre-treatment if recorded as a pre-treatment test or if taken within 7 days of this test. DSTs were classified as taken during 1^st^ line treatment if taken >7 days and up to 9 months after the pre-treatment test. All matches and classification as “pre-treatment” or “treatment” were manually verified.

### Data analysis

Cases were allocated to algorithm based on the algorithm in place at the facility at the time-point i.e. we used an intention to treat approach in the analysis. We compared the proportion of TB cases with DST undertaken and MDR-TB diagnosed pre-treatment in the smear/culture-based algorithm and the Xpert-based algorithm. Among cases without MDR-TB diagnosed pre-treatment, we assessed the proportion with DST undertaken and MDR-TB diagnosed during the course of 1^st^-line TB treatment in both algorithms. Analyses were done for new and for previously treated TB cases.

Descriptive data are presented using proportions and frequencies. Normally distributed continuous outcomes were analysed using the t-test and categorical outcomes using the chi-squared test. A binomial regression model, adjusted for clustering at 60 primary health care sites, was used to estimate differences in the proportions of DST undertaken and MDR-TB cases diagnosed between algorithms and the interaction of age, gender and treatment category variables. We undertook a sub-analysis for new and previously treated cases. We did not consider a time-effect, as the change in the proportion with DST undertaken over time was inherent to the phased introduction of the Xpert-based algorithm. All analyses were undertaken using STATA 13 (StataCorp).

### Ethics statement

The Health Research Ethics Committee at Stellenbosch University (IRB0005239) (N10/09/308) and Ethics Advisory Group at The International Union Against Tuberculosis and Lung Disease (59/10) approved the study. A waiver of informed consent was granted for the use of routine data. The City Health Directorate, Western Cape Health Department and National Health Laboratory Service granted permission to use routine health data. Data sharing is possible following approval by these authorities.

## Results

A total of 5,019 TB cases were diagnosed in the smear/culture-based algorithm and 5,444 in the Xpert-based algorithm. Among these, 7 and 3 cases were excluded from respective algorithms as they were known MDR/XDR-TB cases (with tests incorrectly reported as pre-treatment) and 97 and 72 from respective algorithms were merged with corresponding records as they belonged to individuals diagnosed at multiple facilities. The remaining 4,915 TB cases in the smear/culture group and 5,369 in the Xpert group were included in the analysis.

### Characteristics of TB cases and those with DST undertaken by algorithm

There were no significant differences in age, gender or TB category (new or previously treated) between TB cases in the smear/culture and Xpert groups (Table 1). For those with DST undertaken pre-treatment, cases in the smear/culture group were slightly older and contained a lower proportion of new cases (45% compared to 65% in the Xpert group). Among those with DST undertaken on treatment, the smear/culture group contained a higher proportion of females (43% compared to 32% in the Xpert group).

**Table 1:** Characteristics of TB cases evaluated by algorithm. The smear/culture and Xpert groups were compared using chi-squared tests. Missing values are not shown but have been included in the calculation of percentages. These have been excluded when comparing groups and calculating p-values. Abbreviations: TB–tuberculosis; DST–drug susceptibility test; SD–standard deviation.

		Smear/culture-based algorithm	Xpert-based algorithm	p-value
TB cases	Total	4,915	5,369	-
	Mean age (Years) [SD]	35 [13]	35 [12]	0.905
	Number male (%)	2,702 (55)	2,995 (56)	0.811
	Number female (%)	2,086 (42)	2,290 (43)	
	Number new cases (%)	2,990 (61)	3,322 (62)	0.309
	Number previously treated cases (%)	1,490 (30)	1,583 (29)	
DST undertaken pre-treatment	Total	2,099	4,235	-
	Mean age (Years) [SD]	36 [11]	35 [12]	0.043
	Number male (%)	1,165 (56)	2,396 (57)	0.784
	Number female (%)	879 (42)	1,781 (42)	
	Number new cases (%)	946 (45)	2,733 (65)	<0.001
	Number previously treated cases (%)	1015 (48)	1,253 (30)	
DST undertaken for cases on 1^st^ line TB treatment	Total	221	247	-
	Mean age (Years) [SD]	35 [12]	37 [11]	0.135
	Number male (%)	122 (55)	164 (66)	0.013
	Number female (%)	96 (43)	80 (32)	
	Number new cases (%)	102 (46)	122 (49)	0.469
	Number previously treated cases (%)	101 (46)	105 (43)	

### Proportion of TB cases with drug susceptibility testing and MDR-TB diagnosed by algorithm

DSTs were undertaken pre-treatment for 42.7% of TB cases in the smear/culture group compared to 78.9% in the Xpert group and 5.5% and 7.7% of TB cases in respective groups had MDR-TB diagnosed (Table 2). Among cases not initially diagnosed with MDR-TB, 4.8% and 5.0% in respective groups had DST undertaken during the course of 1^st^-line TB treatment and 0.6% and 0.9% respectively were diagnosed with MDR-TB. In these MDR-TB cases, a lower proportion of cases had received an initial pre-treatment DST in the smear/culture group (5/28) than in the Xpert group (21/43)(p = 0.008).

**Table 2:** Drug susceptibility testing and MDR-TB cases diagnosed by TB diagnostic algorithm. Data is not shown for cases with TB category “unknown” (435 cases (8.9%) in smear/culture group and 464 (8.6%) in Xpert group) but can be calculated based on the numbers shown. DST (drug susceptibility tests) and MDR-TB (multidrug- resistant tuberculosis) pre-treatment are expressed as a percentage of TB cases. DST and MDR-TB on 1^st^ line TB treatment are expressed as a percentage of TB cases not initially diagnosed with MDR-TB. Abbreviations: TB–tuberculosis; MDR-TB—multidrug-resistant tuberculosis.

	Smear/culture-based algorithm Number of cases (%)			Xpert-based algorithm Number of cases (%)		
	All TB cases	New TB cases	Previously treated cases	All TB cases	New TB cases	Previously treated cases
Number of TB cases	4915	2990	1490	5369	3322	1583
DST undertaken pre-treatment	2099 (42.7)	946 (31.6)	1015 (68.1)	4235 (78.9)	2733 (82.3)	1253 (79.2)
MDR-TB diagnosed pre-treatment	269 (5.5)	82 (2.7)	158 (10.6)	415 (7.7)	184 (5.5)	182 (11.5)
DST undertaken on 1^st^ line TB treatment	221 (4.8)	102 (3.5)	101 (7.6)	247 (5.0)	122 (3.9)	105 (7.5)
MDR-TB diagnosed on 1^st^ line TB treatment	28 (0.6)	9 (0.3)	18 (1.4)	43 (0.9)	15 (0.5)	20 (1.4)
Total MDR-TB diagnosed	297 (6.0)	91 (3.0)	176 (11.8)	458 (8.5)	199 (6.0)	202 (12.8)

Among new TB cases, DSTs were undertaken pre-treatment for 31.6% of TB cases in the smear/culture group compared to 82.3% in the Xpert group and 2.7% and 5.5% of new TB cases in respective groups had MDR-TB. Among new TB cases not initially diagnosed with MDR-TB, similar proportions had DST undertaken (3.5% and 3.9% respectively) and were diagnosed with MDR-TB (0.3% and 0.5% respectively) during the course of 1^st^-line TB treatment.

Among previously treated TB cases, DSTs were undertaken pre-treatment for 68.1% of TB cases in the smear/culture group compared to 79.2% in the Xpert group and 10.6% and 11.5% of previously treated TB cases in respective groups had MDR-TB diagnosed. Among previously treated TB cases not initially diagnosed with MDR-TB, similar proportions had DST undertaken (7.6% and 7.5% respectively) and were diagnosed with MDR-TB (1.4% in both algorithms) during the course of 1^st^-line TB treatment.

### Comparing TB cases with DST undertaken pre-treatment and MDR-TB diagnosed by algorithm

The probability of having a DST undertaken pre-treatment was 1.82 times higher in the Xpert group than in the smear/culture group (p<0.001)(Table 3). Previously treated TB cases were more likely to have DST undertaken (RR = 1.09, p = 0.001) than new cases.

**Table 3:** Comparison of TB cases with DST undertaken pre-treatment and MDR-TB diagnosed. The table shows outputs from binomial regression models for all TB cases with drug susceptibility tests (DST) undertaken pre-treatment and multidrug-resistant tuberculosis (MDR-TB) diagnosed and for sub-categories of new and previously treated cases. Facility level clustering has been taken into account in the binomial regression models. Abbreviations: TB–tuberculosis; MDR-TB—multidrug-resistant tuberculosis

	Variable	Risk ratio	Standard error	95% CI	p-value
All TB cases					
DST undertaken	Xpert-based algorithm	1.82	0.07	1.69 to 1.97	<0.001
	Age	~1.00	<0.01	1.00 to 1.00	0.037
	Female gender	0.98	0.01	0.96 to 1.00	0.052
	Previously treated category	1.09	0.03	1.03 to 1.14	0.001
	Constant	0.46	0.02	0.41 to 0.50	<0.001
MDR-TB diagnosed	Xpert-based algorithm	1.42	0.13	1.19 to 1.70	<0.001
	Age	0.99	<0.01	0.99 to <1.00	0.008
	Female gender	1.09	0.10	0.91 to 1.30	0.335
	Previously treated category	2.67	0.27	2.19 to 3.25	<0.001
	Constant	0.05	0.01	0.03 to 0.06	<0.001
New TB cases					
DST undertaken	Xpert-based algorithm	2.64	0.19	2.29 to 3.05	<0.001
	Age	1.00	<0.01	1.00 to 1.00	0.129
	Female gender	0.97	0.02	0.94 to 1.00	0.091
	Constant	0.33	0.03	0.28 to 0.38	<0.001
MDR-TB diagnosed	Xpert-based algorithm	2.09	0.33	1.53 to 2.84	<0.001
	Age	0.99	<0.01	0.98 to <1.00	0.088
	Female gender	1.03	0.15	0.78 to 1.36	0.821
	Constant	0.04	<0.01	0.02 to 0.05	<0.001
Previously treated TB cases					
DST undertaken	Xpert-based algorithm	1.15	0.03	1.10 to 1.21	<0.001
	Age	1.00	<0.01	1.00 to 1.00	0.024
	Female gender	0.99	0.02	0.94 to 1.03	0.553
	Constant	0.76	0.03	0.70 to 0.83	<0.001
MDR-TB diagnosed	Xpert-based algorithm	1.09	0.11	0.90 to 1.34	0.376
	Age	0.99	<0.01	0.98 to 1.00	0.047
	Female gender	1.15	0.13	0.91 to 1.43	0.237
	Constant	0.14	0.03	0.10 to 0.20	<0.01

The likelihood of being diagnosed with MDR-TB was 1.42 times higher in the Xpert group than in the smear/culture group (p<0.001). Previously treated cases were more likely to be diagnosed with MDR-TB pre-treatment than new cases (RR = 2.67, p<0.001).

Among new TB cases, the likelihood of a DST being undertaken pre-treatment was 2.64 times higher (p<0.001) and of MDR-TB being diagnosed was 2.09 times higher (p<0.001) in the Xpert group than in the smear/culture group. Among previously treated TB cases, the likelihood of a DST being undertaken pre-treatment was 1.15 times higher in the Xpert group than in the smear/culture group (p<0.001); however, the risk of being diagnosed with MDR-TB was not increased (RR = 1.09, p = 0.376).

### Comparing cases with DST undertaken whilst on 1st line TB treatment by algorithm and MDR-TB diagnosed

Among TB cases not initially diagnosed with MDR-TB, there was no significant difference in the proportion with DST undertaken during the course of 1^st^-line TB treatment in the Xpert group (RR = 1.02; p = 0.848) compared to with smear/culture group (Table 4). Females were less likely to be screened for drug susceptibility than males (RR = 0.77; p = 0.015). Previously treated TB cases were more likely to be screened for drug susceptibility than new cases (RR = 2.02, p<0.001).

**Table 4:** Comparison of TB cases with DST undertaken whilst on 1^st^ line TB treatment and MDR-TB diagnosed. This table shows outputs from a binomial regression model for all TB cases with drug susceptibility tests (DST) undertaken and for multidrug-resistance tuberculosis (MDR-TB) diagnosed during the course of 1^st^-line TB treatment. Facility level clustering has been taken into account in the models.

	Variable	Risk ratio	Standard error	p-value	95% CI
DST undertaken	Xpert-based algorithm	1.02	0.13	0.848	0.80 to 1.31
	Age	1.00	<0.01	0.760	0.99 to 1.00
	Female gender	0.77	0.08	0.015	0.62 to 0.95
	Previously treated cases	2.02	0.25	<0.001	1.59 to 2.58
	Constant	0.04	<0.01	<0.001	0.03 to 0.06
MDR-TB diagnosed	Xpert-based algorithm	1.12	0.34	0.678	0.63 to 2.03
	Age	0.99	<0.01	0.435	0.98 to 1.01
	Female gender	0.84	0.22	0.520	0.50 to 1.41
	Previously treated cases	3.86	0.84	<0.001	2.52 to 5.90
	Constant	<0.01	<0.01	<0.001	<0.01 to 0.01

The likelihood of being diagnosed with MDR-TB during the course of 1^st^-line TB treatment was similar in the Xpert (RR = 1.12; p = 0.678) and smear/culture groups. Previously treated TB cases were 3.96 times more likely to be diagnosed with MDR-TB than new cases (p <0.001).

## Discussion

Previously treated TB cases are at significantly higher risk of MDR-TB than new cases [11–13]. Many countries including South Africa have historically rationed the use of DST in favour of these cases. Thus, in the previous smear/culture-based algorithm drug-susceptibility testing in presumptive TB cases was limited to those at high risk of MDR-TB, namely those who were previously treated for TB, with MDR-TB contacts or from congregate settings. The assumption was that the smaller proportion of new TB cases with drug resistance would be detected during the course of 1^st^-line treatment.

In comparison, the Xpert-based algorithm required all presumptive TB cases to be screened with Xpert. As expected, our study found that a higher proportion TB cases were screened for drug susceptibility pre-treatment in the Xpert-based algorithm (78.9%) compared to in the smear/culture-based algorithm (42.7%). Overall, TB cases were 82% more likely to be screened for drug susceptibility and 42% more likely to be diagnosed with MDR-TB pre-treatment in the Xpert-based algorithm than in the smear/culture-based algorithm. These differences were accentuated among new cases (RR = 2.64 for DST and RR = 2.09 for MDR-TB). Although previously treated cases were also more likely to have a DST in the Xpert-based algorithm (RR = 1.15) there was no significant increase in the likelihood of an MDR-TB diagnosis.

Interestingly, similar proportions of cases were screened for drug susceptibility (RR = 1.02; p = 0.848) and identified with MDR-TB (R = 1.12; p = 0.678) during the course of 1^st^-line TB treatment in the Xpert-based algorithm compared to in the smear/culture-based algorithm. The assumption that new cases with MDR-TB that were initially missed in the smear/culture-based algorithm would be picked up during the course of treatment appears not to hold true. We speculate that there may have been cases that died or were lost to follow-up before the opportunity to screen them for drug resistance presented.

It is notable that women were less likely to have a DST undertaken during the course of 1^st^ line TB treatment than men. It is difficult to know whether this is due to reduced perceptions of risk (perhaps due to better adherence to treatment) or to women receiving a poorer quality of service [19–23].

There were over double the number of new compared to previously treated TB cases in the Xpert-based algorithm. Although the proportion of MDR-TB is higher among previously treated TB cases than among new cases, the large number of new TB cases means that they contributed almost equally to the number of MDR-TB cases diagnosed pre-treatment with Xpert. Failure to identify these cases early has serious consequences. It contributes to ongoing transmission of MDR-TB which is substantially more expensive [24] and difficult to treat successfully [25]. Inappropriate treatment leads to amplification of drug resistance [26,27] and exacerbates costs [24] and poor treatment outcomes [25]. The direct impact on patients of failing to identify MDR-TB pre-treatment should not be under-estimated. Interviews undertaken as part of PROVE IT illustrated the physical and emotional suffering endured by patients when 1^st^ line TB regimens failed, how socio-economic difficulties were compounded and the devastating impact on families, particularly when children were infected [28,29].

### Strengths and limitations

The strength of an operational evaluation is that it reflects the reality of routine practice, including the impact of inconsistent implementation of diagnostic procedures. The evaluation was undertaken in the early phase of Xpert implementation which may have contributed to sub-optimal implementation of this algorithm, as not all presumptive TB cases received an Xpert test as required, resulting in an under-estimation of effect. However, even in the well-entrenched smear/culture-based algorithm, fewer previously treated cases had DST undertaken as required than in the newly introduced Xpert-based algorithm.

A major strength of the study was that we were had retrospective data that enabled us to assess DST undertaken during the course of treatment. This allowed us to assess whether limited initial DST and MDR-TB diagnosis was compensated by screening and diagnosis during the course of 1^st^-line TB treatment; we found this not to be the case.

The completeness of routine data, with missing age (<5%), gender (<3%) and treatment category (<9%) variables in both groups is a limitation but is unlikely to have influenced overall findings at these levels. We were unable to analyse the interaction of HIV as this data was not available in the electronic laboratory dataset.

Our case definitions did not take account of discordant Rif-R results. Among the 223 cases with Rif-R on Xpert, 24 did not have confirmatory LPA and 12 had a discordant first LPA result (the majority of these did not have further tests undertaken). This may have resulted in an over-estimation of the proportion of true positive Rif-R cases in the Xpert-based algorithm. An intention to treat approach was adopted with the analysis based on the algorithm in place at the facility and not the tests undertaken. During the transition between algorithms, some contamination occurred with facilities in the smear/culture based algorithm testing patients via Xpert, resulting in a slight over-estimation of pre-treatment DST undertaken (additional 102 cases) and MDR-TB cases diagnosed (additional 3 cases).

### Implications for policy and practice

Overall 8.5% of TB cases were detected with MDR-TB in the Xpert-based algorithm compared to 6% in the smear/culture-based algorithm. If one applies these proportions to the approximately 15,000 bacteriologically diagnosed PTB cases in all eight sub-districts in Cape Town annually, it translates to a substantial programmatic effect–an additional 375 MDR-TB cases diagnosed annually.

A recent national DR-TB prevalence survey in South Africa reported high rates of rifampicin resistance—3.4% among new cases and 7.1% among previously treated cases in 2012–2014 [30], confirming high rates of transmission and a substantial national burden. The use of Xpert MTB/RIF as a screening tool for all presumptive TB cases is important in detecting these cases and reducing transmission, but comes at a high cost. A laboratory costing study undertaken as part of PROVE-IT reported an incremental cost-effectiveness ratio of $6,274 per MDR-TB case identified in the Xpert-based algorithm [31].

In addition to identifying patients with MDR-TB, reduced transmission will also be influenced by the proportion who initiate treatment, treatment delay and treatment success. Routine data from South Africa shows that only 62% of detected MDR-TB cases (11,538/18,734) initiated treatment in 2014 [1]. Whilst studies have reported reductions in delay from MDR-TB diagnosis to treatment [32,33], pre-diagnostic delays are unknown. Two systematic reviews in TB patients reported average (mean or median) time delays from symptom onset to first health care visit of 5–162 days [34] and 7–69 days [35] respectively. These may well be longer for MDR-TB patients. Treatment success rates are dismal: MDR-TB treatment success rates of 39% were reported for cases registered nationally in 2009–2011 [25). Decentralised treatment models also reported low success rates: 52% in a study in the Western Cape [36] and in Kwa-Zulu Natal, 54% from centralised treatment compared to 58% from decentralised treatment [37]. Health system improvements, appropriate support for patients and improved regimens are required to improve treatment initiation, adherence and outcomes in order to help reduce MDR-TB transmission.

## Conclusion

Universal drug susceptibility testing (i.e. among all presumptive TB cases) in the Xpert-based algorithm detected a significantly higher proportion of MDR-TB cases than the rationed approach used in the smear/culture-based algorithm. The previous strategy of only screening new TB cases when 1^st^ line treatment failed did not compensate for cases missed pre-treatment. However, universal screening with Xpert is an expensive strategy–in order to justify this expenditure, patient and diagnostic delays must be decreased; health services strengthened and new treatment regimens introduced to improve MDR-TB treatment initiation and outcomes. The cost of Xpert needs to be reduced to ensure sustainability.
